# Modeling the Distribution of the Chytrid Fungus *Batrachochytrium dendrobatidis* with Special Reference to Ukraine

**DOI:** 10.3390/jof9060607

**Published:** 2023-05-25

**Authors:** Volodymyr Tytar, Oksana Nekrasova, Mihails Pupins, Arturs Skute, Muza Kirjušina, Evita Gravele, Ligita Mezaraupe, Oleksii Marushchak, Andris Čeirāns, Iryna Kozynenko, Alena A. Kulikova

**Affiliations:** 1 I.I. Schmalhausen Institute of Zoology, National Academy of Sciences of Ukraine, 01030 Kyiv, Ukraine; vtytar@gmail.com (V.T.); oneks22@gmail.com (O.N.); kozinenko@gmail.com (I.K.); 2 Department of Ecology, Institute of Life Sciences and Technologies, Daugavpils University, LV5400 Daugavpils, Latvia; mihails.pupins@gmail.com (M.P.); arturs.skute@du.lv (A.S.); muza.kirjusina@du.lv (M.K.); evita_22@inbox.lv (E.G.); ligita.kecko@gmail.com (L.M.); cuskisa@gmail.com (A.Č.); 3 Independent Researcher, LV5400 Daugavpils, Latvia; elen.kulikova@gmail.com

**Keywords:** amphibia, fungal diseases, spreading, infection, pathogen, GIS modeling, species distribution modelling

## Abstract

Amphibians are the most threatened group of vertebrates. While habitat loss poses the greatest threat to amphibians, a spreading fungal disease caused by *Batrachochytrium dendrobatidis* Longcore, Pessier & D.K. Nichols 1999 (Bd) is seriously affecting an increasing number of species. Although Bd is widely prevalent, there are identifiable heterogeneities in the pathogen’s distribution that are linked to environmental parameters. Our objective was to identify conditions that affect the geographic distribution of this pathogen using species distribution models (SDMs) with a special focus on Eastern Europe. SDMs can help identify hotspots for future outbreaks of Bd but perhaps more importantly identify locations that may be environmental refuges (“coldspots”) from infection. In general, climate is considered a major factor driving amphibian disease dynamics, but temperature in particular has received increased attention. Here, 42 environmental raster layers containing data on climate, soil, and human impact were used. The mean annual temperature range (or ‘continentality’) was found to have the strongest constraint on the geographic distribution of this pathogen. The modeling allowed to distinguish presumable locations that may be environmental refuges from infection and set up a framework to guide future search (sampling) of chytridiomycosis in Eastern Europe.

## 1. Introduction

Amphibians are the most sensitive group of vertebrates—41% of currently known species are threatened with extinction [1,2,3]. Although habitat loss clearly poses the greatest threat to amphibians, a fungal disease threat (*Batrachochytrium dendrobatidis* Longcore, Pessier & D.K. Nichols 1999) has affected an increasing number of species [4]. This disease caused by the chytrid fungus *B. dendrobatidis* (Bd) has been linked to the declines in amphibian species globally and represents the greatest documented loss in biodiversity attributable to a pathogen [5]. A hypervirulent and globally distributed pandemic lineage (*B. dendrobatidis*-GPL) is considered the leading cause of population reduction in amphibians [6]. 

The Bd fungus has a wide geographical and host range in Europe; however, there currently is still a lack of complete understanding regarding the potential impacts of the amphibian communities. As of now, it has been registered in almost all Anura species in 26 European countries [7]. The susceptibility of various amphibian species across Europe is still not fully understood, but declines in populations of species such as the common midwife toad (*Alytes obstetricans* (Laurenti, 1768)) and the common toad (*Bufo bufo* (Linnaeus, 1758)) [8]. In other species in which the fungus presence has been confirmed, infection with Bd does not always result in disease development, which could explain why mass die-offs of amphibians such as those observed in other parts of the world have not been seen [9]. Some species of anurans have limited immunity to Bd, while others such as water frogs (*Pelophylax* spp.) exhibit tolerance [10,11]. Some research also showed that populations of the yellow-bellied toad (*Bombina variegata* (Linnaeus 1758)) can coexist with Bd. However, the future safety of these toads may be compromised due to the potential effects of climate change and other environmental factors. The absence of widespread die-offs in many areas of Europe may be attributed to the fact that Bd has multiple strains with varying levels of virulence [12].

Although Bd is widely prevalent, there are identifiable heterogeneities in the pathogen’s distribution that have been linked to environmental parameters [12]. In this respect, species distribution models (SDMs) have proven to be useful tools for predicting Bd distribution and elucidating the importance of a wide range of environmental covariates considered to affect Bd occurrence. Based on published data on *B. dendrobatidis* occurrence, SDMs can be used to evaluate the factors affecting its occurrence and predict its distribution [13,14,15]. The first developed Bd SDMs were global in scope [16,17].

It is well known that one major assumption of SDMs is that the species being modeled is in equilibrium with its environment. Considering invasive species, their distribution today may not be in equilibrium with current environmental conditions, and it may take centuries or millennia for invasive species to stabilize. However, global or other large-scale SDMs may provide overarching clues to Bd climate niche characteristics, and downscaled models such as those at the continental scale show that different climate metrics can be important predictors of Bd occurrence at finer spatial scales [17]. By considering geographically more restricted areas such as Ukraine or Eastern Europe, we anticipate obtaining a better view on Bd niche requirements that will aid us in reaching our objectives. Nevertheless, despite this reservation, habitat suitability models have been shown to be highly predictive [18,19].

Early detection and rapid response to incoming aliens are required for a successful management response [20]. Early-response strategies involve surveying risk areas under threat of invasion. SDMs help to identify such areas at risk that are suitable for the establishment of alien species, for instance by matching suitable climate conditions [21]. Identifying areas where a species is more likely to occur can also be used to guide sampling protocols and prioritizing areas of study [22]. Our objectives were to:

(1) Identify priority survey areas in Eastern Europe (with a special focus on Ukraine) where future outbreaks of Bd could occur (“hotspots”), but perhaps what was equally important was the recognition of locations that may be environmental refuges (“coldspots”) from infection (especially for amphibians that have certain sensitive conservation status) [23];
(2) Identify bioclimatic and other environmental conditions that constrain the geographic distribution of this pathogen in the study area.

It is necessary to mention that global or other large-scale SDMs may provide overarching clues to Bd climate niche characteristics, but downscaled models such as those at the continental scale show that different climate metrics can be important predictors of Bd occurrence at finer spatial scales. Hence, the scale of Ukraine or Eastern Europe is a much finer spatial scale than previously addressed by those recent large models, which makes this study exactly the next needed step.

Undoubtedly, recognizing both “hot-” and “coldspots” are essential for proactive conservation planning [6].

## 2. Materials and Methods

As input, standard distribution models (SDMs) require georeferenced biodiversity observations. The globally dominant lineage is the pandemic one, while other lineages are of limited distribution or known transmission routes [24]. Therefore, this study did not take into account the lineage identity of positive samples for which there is yet insufficient data. Additionally, the lineages of Bd are not well mapped, resulting in a lack of necessary data for analysis. Localities for Bd were gathered from GBIF [25] and the literature [26,27,28,29,30,31,32,33,34,35]. Using quantitative PCR as described in [36], 20 new records of Bd from Belarus were added to the unpublished database of (A. A. Kulikova. To account for sampling bias, we used the nearest neighbor distance method (‘ntbox’ package in R; Osorio-Olvera et al., 2020) for thinning the data [37].

Because many limitations are associated with SDM projections, particularly when it comes to building an SDM for a species expanding its home range in a new area [38], we employed for the analysis only records of European localities. In addition, a global description of the niche does not account for the specificities of local invasive ranges (local environment, local biotic interactions, and specific human uses) [39]. Studies using records solely from the invasive range have demonstrated the ability to make adequate predictions regarding the expansion of invasive alien species [40,41,42].

SDMs commonly utilize associations between environmental variables and known species-occurrence records to identify environmental conditions within which populations can be maintained. SDMs extrapolate in situ habitats to identify geographic regions that have similar combinations of these values [43]. SDMs may be primarily climate-driven, meaning that the variables used to develop them typically portray climatic factors [44]. In this study, several types of environmental variables besides climatic at a geodetic resolution of 2.5 arc minutes were used as proxies for the fundamental niche [45].

First, 19 bioclimatic variables were downloaded from the WorldClim website (http://www.worldclim.com/version2 (accessed on 12 November 2022)) indicating a general trend of precipitation and temperature as well as extremity and seasonality of temperature [46]. However, we excluded four variables (bio8, bio9, bio18, and bio19) owing to their known spatial artifacts when following the protocol implemented in previous similar studies [47,48]. Other “quarter” variables were removed as well because they were highly correlated with monthly values and largely carried redundant information. 

Second, our model included a set of 16 climate and two topographic variables (the ENVIREM dataset downloaded from http://envirem.github.io (accessed on 20 January 2023); Table 1), many of which were likely to have direct relevance to ecological or physiological processes determining species distributions [49,50,51,52]. The included topographic variables were potentially important as well because they could modify the effect of the climate descriptors. 

Third, topography as measured by elevation and its derived variables (e.g., slope and aspect) was key for characterizing spatial heterogeneity and the abiotic environment in a given area, subsequently driving hydrological, geomorphological, and biological processes. All developed variables were available for download and visualization at the EarthEnv project site (http://www.earthenv.org/topography (accessed on 21 January 2023)) [52].

Fourth, metrics of land cover were included in our models [53] (Table 1). Land cover information offers a powerful first-order proxy for locally expected biodiversity and ecological processes [54]. Land cover is also considered relevant in models aimed at predicting species distributions because it adds realistic information on habitat fragmentation and human influence, which are not represented in more commonly used sets of climatic variables [55]. Consensus information on the prevalence of 12 land cover classes across the globe was downloaded from the EarthEnv project site (https://www.earthenv.org/landcover (accessed on 25 January 2023)) [56].

Finally, soil variables were included in our analyses. We assumed that the integration of soil factors in SDMs could help improve our understanding of factors that limit the Bd distribution range. These comprised 9 layers of soil physical and chemical properties recommended or previously used for building SDMs [57,58,59]. Soil grids were obtained from the Land-Atmosphere Interaction Research Group at Sun Yatsen University [60]. The values of the first five layers (0–49 cm) in the profile were derived and averaged for subsequent modeling.

Predictors often show high collinearity, and most SDM approaches require the selection of one among those strongly correlated [61]. In order to carry out such selection, the ‘removeCollinearity’ function in the ‘virtualspecies’ R (v. 3.3.3) package was employed [62]. This function analyzes the correlation among predictors, and using a 0.7 cut-off [63] returned a vector containing the names of those that were not colinear. It also grouped predictor variables according to their degree of collinearity, so from each such group consisting of two or more variables, those putatively most relevant to Bd could be selected. Because predictor variables are commonly skewed or have outliers, the Spearman correlation method was applied.

Today, a number of modeling approaches are at hand [64] depending on the environmental questions and available data; in particular, presences and absences. However, in the case of an emerging disease, the use of presence-only data is crucial to avoid possible complications due to false negatives and temporal variations in pathogen prevalence [65]. Therefore, a presence-only approach was chosen.

SDMs were generated by employing Bayesian additive regression trees (BARTs), a cutting-edge technique in this field. Running SDMs with BARTs has been substantially facilitated by the development of the R (v. 3.3.3) package ‘embarcadero’ [66], which is highly effective at identifying informative subsets of predictors. The package includes methods for generating and plotting response curves, illustrating the effect of selected variables on habitat suitability. The algorithm computes habitat suitability values ranging from 0 for a fully non-suitable habitat to 1 for a fully suitable habitat.

Then, ‘Boruta’, one of the most effective predictor selection algorithms implemented in R (v. 3.3.3) [67], was used to create a custom-made set of predictors for building a consensus model.

To prepare the input, we used pre-modeling functions from the ‘flexsdm’ R (v. 3.3.3) package [68]. The calibration area was defined using buffers around presence points. Because Bd is effective at dispersing, relatively large 500 km buffers were chosen [27]. Filtering the occurrence data was used to reduce sample bias by randomly removing points where they were dense (oversampling) in the environmental and geographical spaces.

The models were evaluated using the area under the receiver operating characteristic curve (AUC) [44] and the true skill statistic (TSS) [69]. AUC scores range from 0 to 1 (with 0 for systematically wrong model predictions and 1 for systematically perfect model predictions); AUC values 0.7 to 0.8 are considered acceptable, while values > 0.8 are considered to be good to excellent. TSS values range from −1 to +1 (with −1 corresponding to systematically wrong predictions and +1 to systematically correct predictions) [70].

TSS values <0.4 are considered poor, 0.4–0.8 useful, and >0.8 are good to excellent. Because AUC has its drawbacks [71], we employed the continuous Boyce index, which only requires presences and measures how much model predictions differ from random distribution of the observed presences across the prediction gradients [72]. Thus, it is an appropriate metric in the case of presence-only models. It is continuous and varies between −1 and +1. Positive values indicate a model that presents predictions that are consistent with the distribution of presences in the evaluation dataset, values close to zero mean that the model is not different from a random model, and negative values indicate counter predictions [73]. Evaluation metrics were calculated using the script posted by A.M. Barbosa on R-bloggers (https://www.r-bloggers.com/2022/05/model-evaluation-with-presence-points-and-raster-predictions/ (accessed on 7 January 2023)).

For guidance regarding the search for Bd and distinguishing areas in Ukraine where the pathogen most possibly might occur, the consensus distribution model was categorized into three frequency distribution classes (i.e., low, medium, and high) using Jenks’ natural breaks classification, which (like k-means clustering) maximizes the variance between classes while minimizing the variance within classes [74].

We used a 50% habitat suitability threshold [75] as a cut-off above which responses of the most contributive variables could be analyzed in terms of their impact on habitat suitability.

Maps of habitat suitability in the GeoTIFF format were processed and visualized in SAGA GIS (v.2.14) [75]; statistical data was analyzed using the PAST software (v. 4.03) package [76] and/or the R environment (v. 3.3.3) [77].

## 3. Results and Discussion

The update of published and unpublished Bd-occurrence data yielded a total of 234 non-duplicate georeferenced records across Europe. The results of grouping predictor variables according to their degree of collinearity at a cutoff of 0.7 yielded a subset of metrics included in the analyses (Table 1). Variables selected for checking their relevance for Bd are marked with an asterisk.

### 3.1. SDMs Based on Selected WorldClim v.2 Predictors

Using the pre-modeling functions from the ‘flexsdm’ R package, the number of presence records was reduced to 114. The BART model showed a very good performance with a Boyce index of 0.951 and an AUC and TSS of 0.824 and 0.525, respectively.

**Figure 1:**
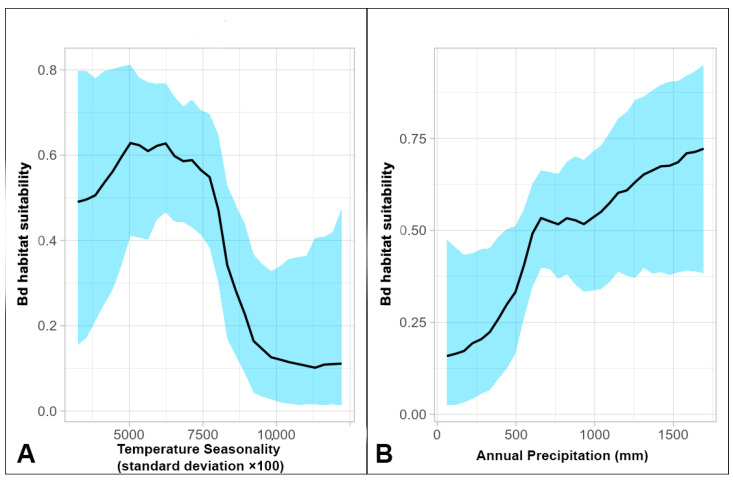
Bd habitat suitability response curves (1.0 corresponds to full association of Bd with *x*-axis metric: (**A**) partial response curve for Temperature Seasonality (SD × 100); (**B**) partial response curve for Annual Precipitation (mm). Response = habitat suitability.

The BART model indicated the importance of Temperature Seasonality, Annual Precipitation, and the Min. Temperature of Coldest Month (Figure 1) in predicting the occurrence of Bd. Temperature Seasonality is a measure (in units of standard deviation (SD)) of temperature variability over the course of a year. Figure 1A depicts that increasing the variability in temperature first established a hump-shaped relationship with habitat suitability within a certain interval (using a 50% threshold, that would be approximately between 4000 and 8000 units) (Figure 1A). Further increasing variability produced a sharp negative effect. Notably, Temperature Seasonality was highly correlated with Temperature Annual Range, a surrogate for ‘continentality’ [78], and in the study area, both exhibited a strong longitudinal gradient with values increasing toward the east. Regarding Annual Precipitation (Figure 1B), rising values were found as low precipitation values increased until reaching the mark of around 600 mm, after which the curve steadily approached precipitation values for which habitat suitability was the highest. Previously, Annual Precipitation was found along with Annual Mean Temperature to highly influence the distribution of Bd [24]. Other studies, in addition to stressing the importance of annual precipitation, reported the optimum rainfall for Bd to be between 1500 and 2500 mm a year [79,80] or at an initial modal maximum of around 1200–1400 mm realistic for the European study area [81]. In any event, the graph in Figure 1B leads to similar conclusions.

**Figure 2:**
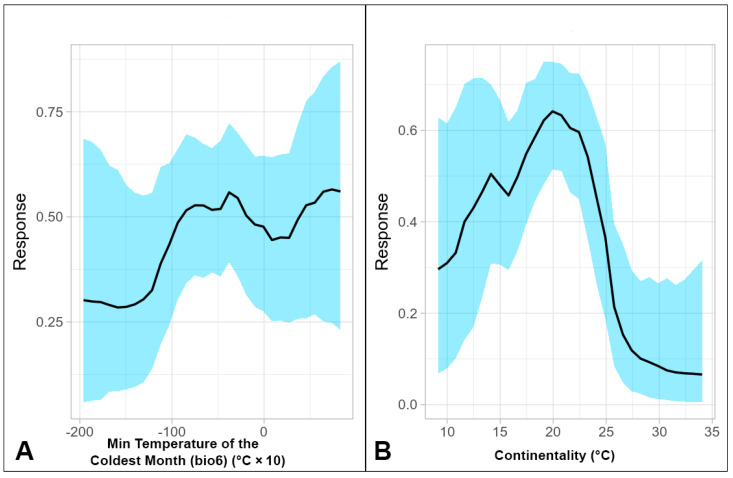
(**A**) Partial response curve for Min. Temperature of Coldest Month (°C × 10); (**B**) partial response curve for Continentality (°C).

In terms of the Min. Temperature of Coldest Month (Figure 2A), habitat suitability remained low before reaching a 50% threshold in a period after −9 °C, which was well below the critical thermal minima of +4 °C [82]. In other places (for instance, the USA (WY, ME, CO, and CA), Bd localities reach the lowest coldest temperatures—down to −19.6 °C [74]. However, the pathogen is hardly exposed to such temperatures because Bd is strongly associated with aquatic habitats and host species that hibernate in aquatic microhabitats rather than terrestrial [83]. Therefore, the fungus is largely buffered from such extreme external conditions.

### 3.2. SDMs Based on Selected ENVIREM Predictors

After performing the pre-modeling functions, the number of presence records was reduced to 161. The model showed a very good performance with a Boyce index of 0.971, AUC = 0.793, and TSS = 0.471.

The BART algorithm distinguished ‘Continentality’ and ‘Annual potential evapotranspiration’ as important predictors. ‘Continentality’ (or the same as Temperature Annual Range from the WorldClim v.2 dataset) is influenced by distance from oceans; i.e., it is a proxy of maritimity and actual Continentality [84]. The response curve for this variable presented in Figure 2B shows a general hump-shaped relationship with habitat suitability (Figure 2B). Using the 50% habitat suitability threshold, suitable conditions for Bd were theoretically found between +17 and +24 °C, after which the response curve sharply declined to a negligible level. Maritimity below +17 °C, as found in much of the Atlantic biogeographical region of Europe [85], appeared unfavorable for the pathogen (likely because of the June–July temperatures), whereas toward the east (roughly beyond the longitude of 26° E) the limiting factor was freezing winter temperatures; this exhibited itself in a profound way as shown by the steeply declining response curve.

**Figure 3:**
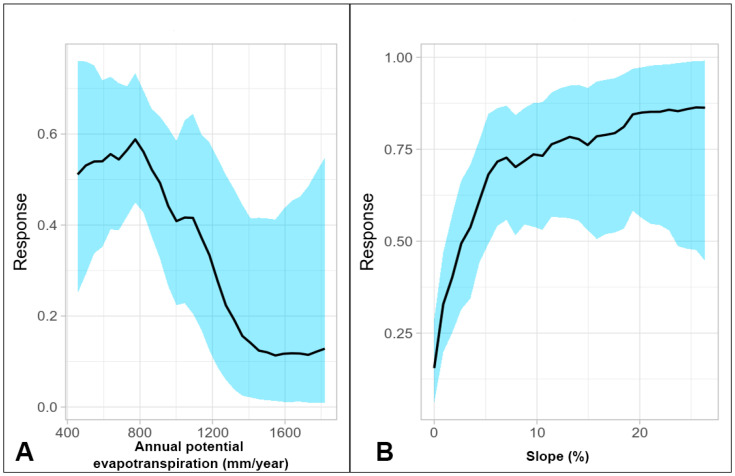
(**A**) Partial response curve for Annual potential evapotranspiration (mm/year); (**B**) partial response curve for slope (%).

‘Annual potential evapotranspiration’ relates to the ability of the atmosphere to remove water through evapotranspiration processes and is strongly influenced by temperature [86]. It is regarded as an index meant to represent available environmental energies and ecosystem productivity [87]. Based on Figure 3A, it can be deduced that suitable conditions for Bd were apparently found (once again using the 50% threshold) between 460 and 900 mm/year, after which the response curve continued to rapidly decline (Figure 3A). Places with high rates of ‘Annual potential evapotranspiration’ appeared to be less suitable for the pathogen. Using the ‘contour lines’ module in SAGA GIS, it could be shown that these areas were located primarily in Southern Europe (below a latitude of approximately 47° N). In a related manner, the minimum monthly potential evapotranspiration was found to be an important driving factor of spatial patterns of amphibian chytridiomycosis [88].

### 3.3. SDMs Based on Selected Topographical Variables from the EarthEnv Dataset

Filtering the number of presence records reduced them to 138. The BART model showed an acceptable performance with the continuous Boyce index reaching 0.778 and an AUC and TSS of 0.766 and 0.396, respectively. The BART algorithm highlighted ‘Slope’ as the most important predictor. It is worth noting here that elevation is amongst the topographical variables repeatedly considered important for shaping the distribution of Bd. Findings in this respect ranged from establishing evidence of a positive correlation of the pathogen occurrence with elevation [89,90] to the negation of any such correlation [91]. In our case, it was notable that the BART algorithm fully excluded ‘elevation’ from the model.

K.M. Kriger and J.-M. Hero (2007) found a greater prevalence of Bd infection among stream-breeding amphibians in Australia and suggested that dissemination of the pathogen is greatly assisted by flowing water linked to slope gradient [92]. This could occur because flagellated zoospores of Bd rarely swim more than 2 cm prior to encysting [85]. Besides that, the fungus prefers cool temperatures [85,92] and thus should grow better in streams rather than in stagnant water bodies. In another study, the odds of being threatened by Bd were found to be five times higher in stream microhabitats [93]. In our case, habitat suitability noticeably increased with ‘slope’ and reached a maximum at around 25–26% (Figure 3B). Interestingly, the results of a special study showed that runoff increased as slope gradient reached a critical point of 25%, then runoff decreased [94].

### 3.4. SDMs Based on Selected Land Cover Variables from the EarthEnv Dataset

**Figure 4:**
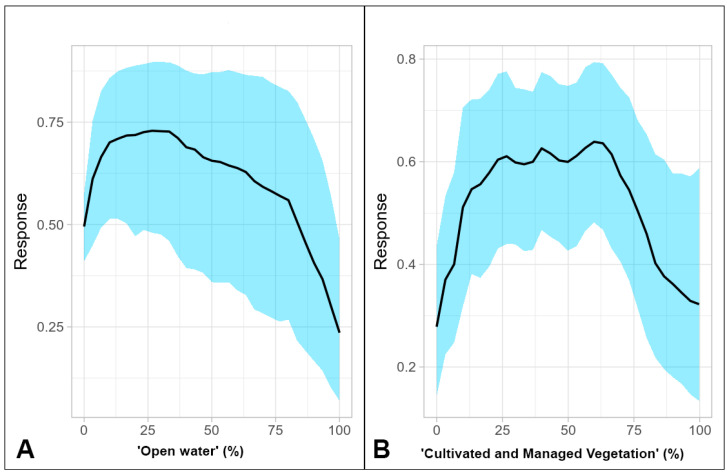
(**A**) Partial response curve for ‘Open water’ (%); (**B**) Partial response curve for ‘Cultivated and Managed Vegetation’ (%).

After filtering, the number of presence records was reduced to 184. The BART model showed a good performance with the continuous Boyce index reaching 0.937 and an AUC and TSS of 0.832 and 0.536, respectively. The BART model strongly highlighted ‘Open water’ and ‘Cultivated and Managed Vegetation’ as important predictors (Figure 4).

Because life cycles of both the pathogen and its hosts are dependent on the availability of water, it was no surprise that the percentage of open water in the landscape was an influential factor. However, in excess it negatively impacted Bd habitat suitability. In this respect, an optimum was reached at around 28%, after which the response curve underwent a gradual decline (Figure 4A). A similar hump-shaped relationship was found between the percentage of land covered with cultivated and managed vegetation; in other words, crop- and farmland and habitat suitability (Figure 4B). Impacts of land-use changes from increased agricultural production are commonly considered negative because they usually alter the habitat physically or chemically such that survival of resident organisms is questionable [95]. This can apply to both Bd and its amphibian hosts. For instance, pesticides can inhibit the immune response in amphibians, making them more susceptible to disease [96], but on the other hand certain fungicides are capable of reducing the number of Bd zoospores on frogs [97].

Nevertheless, initially the increase in the percentage of crop- and farmland in the landscape favored Bd habitat suitability, which apparently was mediated by the hosts while there was still a substantial amount of natural habitat. Amphibians have been found breeding in a variety of habitats that are substantially different from their former natural breeding habitats. In this respect, agricultural landscapes are of no exception [98], and the corresponding human infrastructure has been shown to provide beneficial environments to amphibian species [99,100]. Even so, a further increase of the percentage of crop- and farmland in the landscape (beyond approximately 60%) leads to a sharp drop in habitat suitability (assuming that heavily exploited agricultural areas are not fit for the pathogen).

### 3.5. SDMs Based on Selected Soil Feature Variables from the Land-Atmosphere Interaction Research Group Dataset

**Figure 5:**
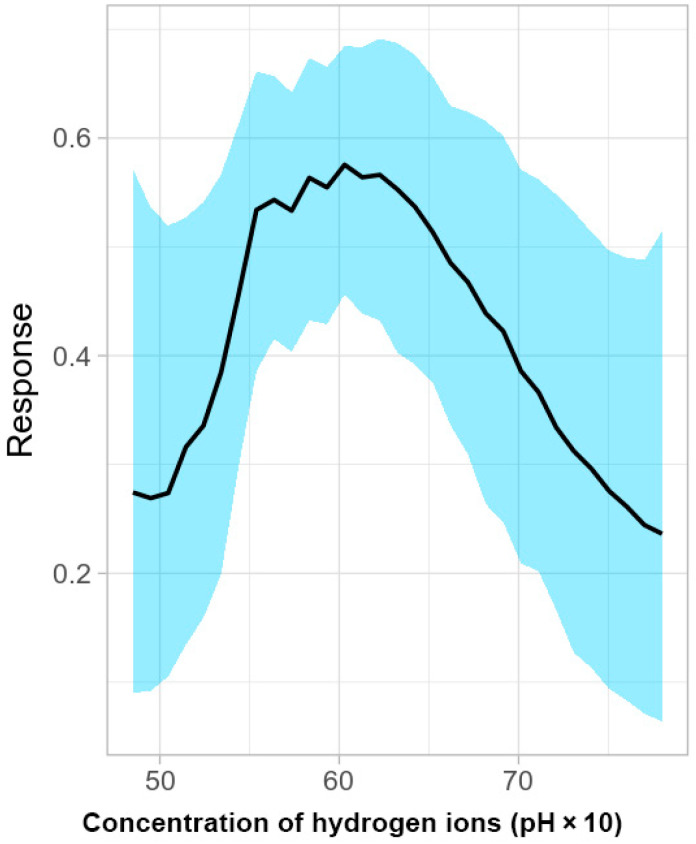
Partial response curve for concentration of hydrogen ions (pH × 10).

Filtering the number of presence records reduced them to 181. The BART model showed a good performance with the continuous Boyce index reaching 0.975 and an AUC and TSS of 0.795 and 0.468, respectively. In terms of top importance, the algorithm pointed toward the concentration of hydrogen ions (pH) (Figure 5).

The partial response curve illustrating the relationship between acidity/basicity and habitat suitability (Figure 5) indicated the best conditions were in the range of pH between 5.5 and 6.5 (with an optimum found around pH 6). This does not mean the pathogen cannot occur in the field beyond this range. If it does, apparently this occurs under less favorable conditions. Experimentally isolates of Bd have been shown to have the most growth at pH 6–7, less growth at pH 8, and minimal growth at pH 4 and 5 [82].

In summary, evaluation metrics for SDMs built on separate environmental datasets showed satisfactory results, meaning the applied predictor variables more or less fully captured habitat characteristics of the fungus species. Perhaps only the model based on topographic variables showed a relatively reduced performance; its evaluation metrics were all lower compared to the rest but nevertheless pointed out the importance of ‘Slope’.

**Figure 6:**
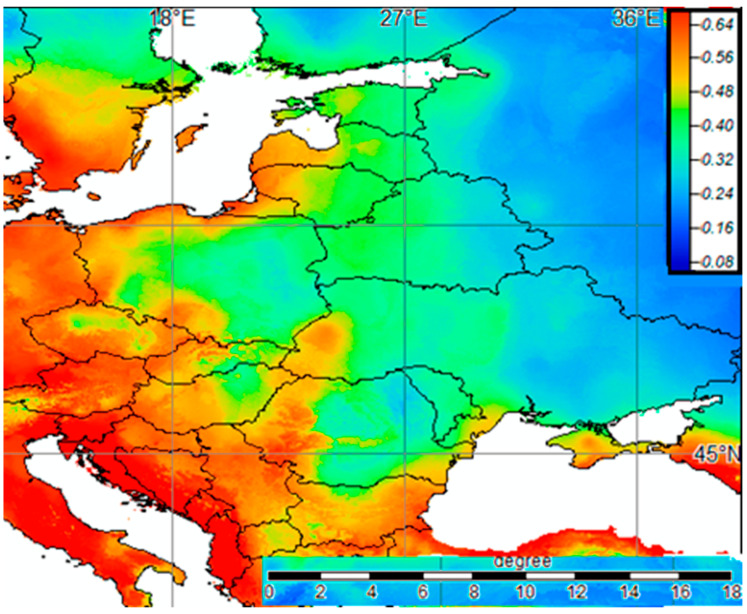
Habitat suitability map for *B. dendrobatidis* in Europe; the legend shows habitat suitability ranging from high (red) to low (blue).

Influential predictors as assessed by the BART algorithm were pooled and subjected to a selection procedure using the R program ‘Boruta’. In the end, the algorithm selected the following variables: Annual Precipitation, Max. Temperature of Warmest Month, Continentality, Gravel content, Organic carbon, PET seasonality, Evergreen/Deciduous Needleleaf Trees, Open Water, Deciduous Broadleaf Trees, Cultivated and Managed Vegetation, and Urban/Built-up. Interestingly, roughly half of these were land cover variables from the EarthEnv dataset. The final BART algorithm with the combined metrics resulted in two top predictors: Continentality and Cultivated and Managed Vegetation. The corresponding SDM (Figure 6) showed a pattern of greater Bd habitat suitability to the west and south of the area modeled.

**Figure 7:**
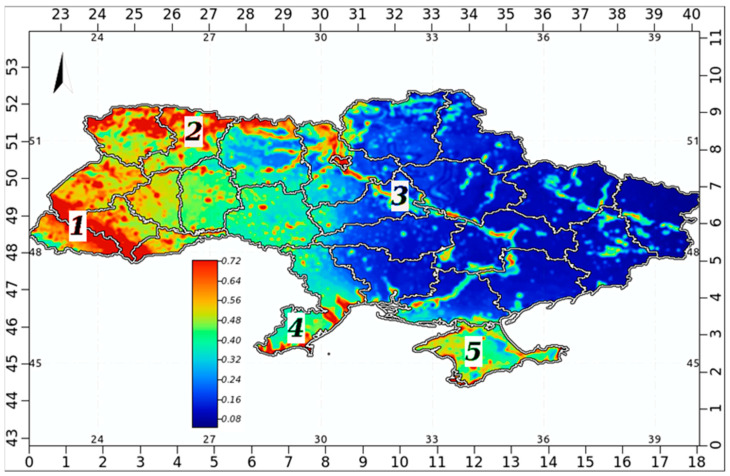
Habitat suitability map for *B. dendrobatidis* in Ukraine; the legend shows habitat suitability ranging from high (red) to low (blue). 1—Carpathian region; 2—forested (Polissia) zone; 3—wetlands along the Dnipro River; 4—wetlands in the Lower Danube area, 5—Crimea.

The downscaled Bd habitat map for Ukraine (Figure 7) similarly showed the greatest Bd habitat suitability to the west with some suitable patches along the Dnipro River, in the Lower Danube area, and in the Crimea. Since the greatest diversity of amphibian species in Ukraine is observed in the Carpathians and forest regions [101], the confirmed presence of Bd tends to be a potential threat to a large proportion of the country’s batrachofauna (17 of 19 species; excluding *Salamandra salamandra* (Linnaeus, 1758)). In addition, suitable patches of habitat for Bd were found along the Dnipro River and in wetlands in the Lower Danube area.

Sporadic areas in the Crimea could accommodate the fungus as well if it ever reaches the peninsula (perhaps via the Northern Crimean irrigation canal).

**Figure 8:**
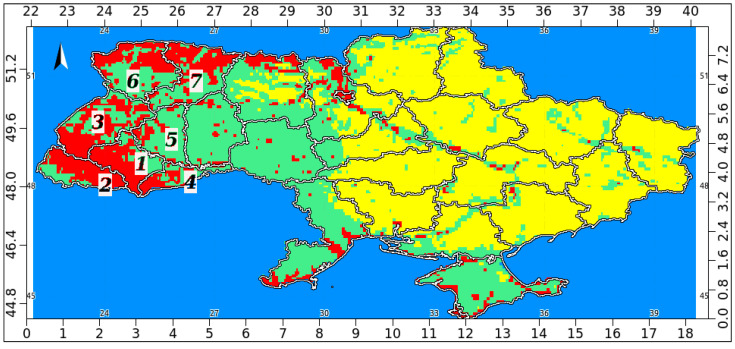
Consensus distribution model categorized into three frequency distribution classes: low (yellow), medium (green), and high (red color). Oblasts: 1—Ivano-Frankivsk; 2—Transcarpathia; 3—L’viv; 4—Chernivtsi; 5—Ternopil; 6—Volyn; 7—Rivne.

A consensus distribution model (Figure 8) was categorized using Jenks’ natural breaks classification into three frequency distribution classes: low (habitat suitability between 0.04 and 0.27), medium (between 0.27 and 0.58), and high (between 0.58 and 0.93). Maximum values of habitat suitability (>0.9) were observed in the following administrative regions (oblasts): Ivano-Frankivsk, Transcarpathia, Lviv, Chernivtsi, Ternopil, Volyn, and Rivne (Figure 8). In summary, regarding the search for Bd, these are areas in Ukraine where the pathogen most possibly might occur, and focusing on them would reduce the areas for direct field samplings and facilitate the identification of potential “hotspots” as well as “coldspots”.

## 4. Conclusions

The continuing spread of Bd suggests that the geographic distribution of this pathogen is greater than currently known. Despite the apparent global invasion of Bd and a corresponding spate of past amphibian losses [84], there are many locations where this disease-causing pathogen has not yet been detected [102]. The use of broad environmental data to model the distribution of such a small fungal organism may cause some uncertainty depending on the geographical and study-variable scales; however, diverse research groups are using these tools to model pathogens’ distributions [22,65,103]. Accordingly, ecological niche modeling (ENM) is an effective way to evaluate how these environmental factors affect current species distributions [104]. In this study, we focused on an attempt to highlight important variables shaping the current niche of a pathogenic organism using a variety of sets of bioclimatic and environmental predictors. The modeling algorithm in this case pointed toward ‘Continentality’ and ‘Cultivated and Managed Vegetation’ as comprehensive predictors of Bd distribution, therefore binding in such a way bioclimatic and human-induced factors. In this respect, unveiling macroscale environmental drivers of Bd and their interactions is crucial for proper conservation management of amphibians in the wake of an expanding disease [105,106]. In addition to the need to identify risk factors facilitating the occurrence of Bd, from a management perspective it is profoundly important to point out high risk areas of infection that potentially can favor the fungus. This is particularly substantial for Ukraine, where surveys for detecting the pathogen are yet to be undertaken.

The results of this study can inform the development of a strategic surveillance and monitoring program for Ukraine amphibian populations and associated threats (including Bd) as well as the development of biosecurity priorities to safeguard unique regions and taxa.

## Figures and Tables

**Table 1:** Groups of intercorrelated variables from the considered environmental datasets.

Groups of Intercorrelated Variables from the WorldClim v.2 Dataset at a Cutoff of 0.7	
Cluster Group	Bioclimatic Variables (Codes)
1.	Temperature Seasonality (bio4) *, Temperature Annual Range (bio7)
2.	Annual Precipitation (bio12) *, Precipitation of Wettest Month (bio13)
3.	Annual Mean Temperature (bio1), Isothermality (bio3), Min. Temperature of Coldest Month (bio6) *
4.	Mean Diurnal Range (bio2), Max. Temperature of Warmest Month (bio5) *
5.	Precipitation of Driest Month (bio14), Precipitation Seasonality (bio15) *
*—Selected variable	
**Groups of Intercorrelated Variables from the ENVIREM Dataset at a Cutoff of 0.7**	
**Cluster Group**	**ENVIREM Variables**
1.	Topographic wetness index *, terrain roughness index
2.	Continentality *
3.	Mean monthly PET^1^ of the wettest quarter *
4.	Growing degree days with mean temperature greater than 0 °C *, growing degree days with mean temperature greater than 5 °C, max. temperature of the coldest month, number of months with mean temp greater than 10 °C, mean monthly PET of coldest quarter, mean monthly PET of driest quarter, thermicity index
5.	Emberger’s pluviothermic quotient, PET seasonality *
6.	Annual PET *, Thornthwaite aridity index, climatic moisture index, min. temp. of the warmest month, mean monthly PET of warmest quarter
*—Selected variable; PET^1^—potential evapotranspiration	
**Groups of Intercorrelated Topographical Variables from the EarthEnv Dataset at a Cutoff of 0.7**	
**Cluster Group**	**Topographical Variables**
1.	Aspect cosine (mean) *
2.	Northness (mean) *
3.	Aspect sine (mean), eastness (mean) *
4.	Topographic position index (mean) *
5.	Roughness (min), slope (min), terrain ruggedness index (min) *
6.	Elevation (min, max, mean *)
7.	Aspect cosine (min, max), aspect sine (min, max), eastness (min, max), eastness (min, max), northness (min, max), roughness (max, mean), slope (max, mean *), topographic position index (min, max), terrain ruggedness index (max, mean)
*—Selected variable	
**Groups of Intercorrelated Land Cover Variables from the EarthEnv Dataset at a Cutoff of 0.7**	
**Cluster Group**	**Land Cover Variables**
1.	Evergreen/Deciduous Needleleaf Trees *
2.	Evergreen Broadleaf Trees *
3.	Deciduous Broadleaf Trees *
4.	Mixed/Other Trees *, Snow/Ice
5.	Shrubs *
6.	Herbaceous Vegetation, Cultivated and Managed Vegetation *
7.	Regularly Flooded Vegetation *
8.	Urban/Built-up *
9.	Barren *
10.	Open Water *
*—Selected variable	
**Groups of Intercorrelated Soil Feature Variables from the Land-Atmosphere Interaction Research Group Dataset at a Cutoff of 0.7**	
**Cluster Group**	**Soil Feature Variables**
1	Bulk density *
2	Cation exchange capacity *
3	Clay content *
4	Gravel content *
5	Organic carbon *
6	pH (H_2_O) *, pH (KCl)
7	Sand content *
8	Silt content *

*—Selected variable.

## Data Availability

The data presented in this study are openly available in [*Batrachochytrium dendrobatidis*. GBIF.org GBIF Occurrence. Available online: https://www.gbif.org/occurrence/download/0215556-230224095556074 (accessed on 5 May 2023)].

## References

[1] Howard S.D., Bickford D.P. (2014). Amphibians over the edge: Silent extinction risk of Data Deficient species. Divers. Distrib..

[2] Catenazzi A. (2015). State of the world’s amphibians. Annu. Rev. Environ. Resour..

[3] IUCN, Conservation International, NatureServe (2008). An Analysis of Amphibians on the 2008 IUCN Red List. www.iucnredlist.org/amphibians.

[4] Stuart S.N., Chanson J.S., Cox N.A., Young B.E., Rodrigues A.S.L., Fischman D.L., Waller R.W. (2004). Status and trends of amphibian declines and extinctions worldwide. Science.

[5] Scheele B.C., Pasmans F., Skerratt L.F., Berger L., Martel A., Beukema W., Acevedo A.A., Burrowes P.A., Carvalho T., Catenazzi A. (2019). Amphibian fungal panzootic causes catastrophic and ongoing loss of biodiversity. Science.

[6] Miller C.A., Tasse Taboue G.C., Ekane M., Robak M., Sesink Clee P.R., Richards-Zawacki C., Fokam E.B., Fuashi N.A., Anthony N.M. (2018). Distribution modeling and lineage diversity of the chytrid fungus *Batrachochytrium dendrobatidis* (Bd) in a central African amphibian hotspot. PLoS ONE.

[7] Allain S.J.R., Duffus A.L.J. (2019). Emerging infectious disease threats to European herpetofauna. Herpetol. J..

[8] Bosch J., Martínez-Solano I. (2006). Chytrid fungus infection related to unusual mortalities of *Salamandra salamandra* and *Bufo bufo* in the Penalara Natural Park, Spain. Oryx.

[9] Berger L., Speare R., Daszak P., Green D.E., Cunningham A.A., Goggin C.L., Slocombe R., Ragan M.A., Hyatt A.D., McDonald K.R. (1998). Chytridiomycosis causes amphibian mortality associated with population declines in the rain forests of Australia and Central America. Proc. Natl. Acad. Sci. USA.

[10] Daum J.M., Davis L.R., Bigler L., Woodhams D.C. (2012). Hybrid advantage in skin peptide immune defenses of water frogs (*Pelophylax esculentus*) at risk from emerging pathogens. Infect. Genet. Evol..

[11] Woodhams D.C., Bigler L., Marschang R. (2012). Tolerance of fungal infection in European water frogs exposed to *Batrachochytrium dendrobatidis* after experimental reduction of innate immune defenses. BMC Vet. Res..

[12] Fisher M.C., Garner T.W.J., Walker S.F. (2009). Global Emergence of *Batrachochytrium dendrobatidis* and amphibian chytridiomycosis in space, time, and host. Annu. Rev. Microbiol..

[13] Rebollar E.A., Hughey M.C., Harris R.N., Domangue R.J., Medina D., Ibáñez R., Belden L.K. (2014). The lethal fungus *Batrachochytrium dendrobatidis* is present in lowland tropical forests of far eastern Panamá. PLoS ONE.

[14] Zimkus B.M., Baláž V., Belasen A.M., Bell R.C., Channing A., Doumbia J., Fokam E.B., Gonwouo L.N., Greenbaum E., Gvoždík V. (2020). Chytrid Pathogen (Batrachochytrium dendrobatidis) in African Amphibians: A Continental Analysis of Occurrences and Modeling of Its Potential Distribution. Herpetologica.

[15] Olson D.H., Aanensen D.M., Ronnenberg K.L., Powell C.I., Walker S.F., Bielby J., Garner T.W.J., Weaver G., Fisher M.C. (2013). Mapping the global emergence of *Batrachochytrium dendrobatidis*, the amphibian chytrid fungus. PLoS ONE.

[16] Rodder D., Kielgast J., Bielby J., Schmidtlein S., Bosch J.T., Garner W.J., Veith M., Walker S., Fisher M.C., Lötters S. (2009). Global amphibian extinction risk assessment for the panzootic chytrid fungus. Diversity.

[17] Liu X., Rohr J.R., Li Y.M. (2013). Climate, vegetation, introduced hosts and trade shape a global wildlife pandemic. Proc. Biol. Sci..

[18] Chai S.-L., Zhang J., Nixon A., Nielsen S. (2016). Using risk assessment and habitat suitability models to prioritise invasive species for management in a changing climate. PLoS ONE.

[19] Xie G.Y., Olson D.H., Blaustein A.R. (2016). Projecting the global distribution of the emerging amphibian fungal pathogen, *Batrachochytrium dendrobatidis*, based on IPCC climate futures. PLoS ONE.

[20] Kaiser B.A., Burnett K.M. (2010). Spatial economic analysis of early detection and rapid response strategies for an invasive species. Resour. Energy Econ..

[21] Srivastava V., Lafond V., Griess V.C. (2019). Species distribution models (SDM): Applications, benefits and challenges in invasive species management. CAB Rev. Perspect. Agric. Vet. Sci. Nutr. Nat. Resour..

[22] Guimarães A., Silva P.H.D., Carneiro F.M., Silva D.P. (2020). Using distribution models to estimate blooms of phytosanitary cyanobacteria in Brazil. Biota Neotrop..

[23] Zumbado-Ulate H., García-Rodríguez A., Vredenburg V.T., Searle C. (2019). Infection with *Batrachochytrium dendrobatidis* is common in tropical lowland habitats: Implications for amphibian conservation. Ecol. Evol..

[24] O’Hanlon S.J., Rieux A., Farrer R.A., Rosa G.M., Waldman B., Bataille A., Kosch T.A., Murray K.A., Brankovics B., Fumagalli M. (2018). Recent Asian origin of chytrid fungi causing global amphibian declines. Science.

[25] *Batrachochytrium dendrobatidis*. GBIF.org GBIF Occurrence. https://www.gbif.org/occurrence/download/0215556-230224095556074.

[26] Greenberg D.A., Palen W.J., Mooers A.Ø. (2017). Amphibian species traits, evolutionary history and environment predict *Batrachochytrium dendrobatidis* infection patterns, but not extinction risk. Evol. Appl..

[27] Schatz A.M., Kramer A.M., Drake J.M. (2017). Accuracy of climate-based forecasts of pathogen spread. R. Soc. Open Sci..

[28] Harmos K., Bosch J., Thumsová B., Martínez-Silvestre A., Velarde R., Voros J. (2021). Amphibian mortality associated with chytridiomycosis in Central-Eastern Europe. Herpetol. Notes.

[29] Saare L., Laasmaa A., Anslan S., Rannap R., Tedersoo L. (2021). Surveying for *Batrachochytrium dendrobatidis* and *B. salamandrivorans* in wild and captive amphibian populations in Estonia and Latvia. Dis. Aquat. Org..

[30] Palomar G., Jakóbik J., Bosch J., Kolenda K., Kaczmarski M., Jośko P., Roces-Díaz J.V., Stachyra P., Thumsová B., Zieliński P. (2021). Emerging infectious diseases of amphibians in Poland: Distribution and environmental drivers. Dis. Aquat. Org..

[31] Balaz V., Vojar J., Civiš P., Šandera M., Rozínek R. (2014). Chytridiomycosis risk among Central European amphibians based on surveillance data. Dis. Aquat. Org..

[32] Baláž V., Vörös J., Civiš P., Vojar J., Hettyey A., Sós E., Dankovics R., Jehle R., Christiansen D.G., Clare F. (2014). Assessing Risk and Guidance on Monitoring of *Batrachochytrium dendrobatidis* in Europe through Identification of Taxonomic Selectivity of Infection. Conserv. Biol..

[33] Kolenda K., Najbar A., Ogielska M., Baláž V. (2017). *Batrachochytrium dendrobatidis* is present in Poland and associated with reduced fitness in wild populations of *Pelophylax lessonae*. Dis. Aquat. Org..

[34] Lastra González D., Baláž V., Vojar J., Chajma P. (2021). Dual Detection of the Chytrid Fungi *Batrachochytrium* spp. with an Enhanced Environmental DNA Approach. J. Fungi.

[35] Kulikova A.A., Pupina A., Pupins M., Čeirāns A., Baláž V. (2022). Survey for *Batrachochytrium dendrobatidis* and *Batrachochytrium salamandrivorans* in Latvian water frogs. J. Wildl. Dis..

[36] Blooi M.F., Pasmans J.E., Longcore A., Spitzen-van der Sluijs A., Vercammen F., Martel A. (2013). Duplex Real-Time PCR for rapid simultaneous detection of *Batrachochytrium dendrobatidis* and *Batrachochytrium salamandrivorans* in amphibian samples. J. Clin. Microbiol..

[37] Osorio-Olvera L., Lira-Noriega A., Soberón J., Townsend Peterson A., Falconi M., Contreras-Díaz R.G., Martínez-Meyer E., Barve V., Barve N. (2020). ntbox: An r package with graphical user interface for modelling and evaluating multidimensional ecological niches. Methods Ecol. Evol..

[38] Bradley B.A., Blumenthal D.M., Wilcove D.S., Ziska L.H. (2010). Predicting plant invasions in an era of global change. Trends Ecol. Evol..

[39] Gallien L., Douzet R., Pratte S., Zimmermann N.E., Thuiller W. (2012). Invasive species distribution models—How violating the equilibrium assumption can create new insights. Glob. Ecol. Biogeogr..

[40] Barbet-Massin M., Rome Q., Villemant C., Courchamp F. (2018). Can species distribution models really predict the expansion of invasive species?. PLoS ONE.

[41] Loo S.E., Nally R.M., Lake P.S. (2007). Forecasting New Zealand mudsnail invasion range: Model comparisons using native and invaded ranges. Ecol. Appl..

[42] Dullinger S., Kleinbauer I., Peterseil J., Smolik M., Essl F. (2009). Niche based distribution modelling of an invasive alien plant: Effects of population status, propagule pressure and invasion history. Biol. Invasions.

[43] Franklin J. (2009). Mapping Species Distributions, Spatial Inference and Prediction.

[44] Kriticos D.J. (2012). Regional climate-matching to estimate current and future sources of biosecurity threats. Biol. Invasions.

[45] Peterson A.T., Soberón J., Pearson R.G., Anderson R.P., Martinez-Meyer E., Nakamura M., Araújo M. (2011). Ecological Niches and Geographic Distributions.

[46] Fick S.E., Hijmans R.J. (2017). WorldClim 2: New 1-km spatial resolution climate surfaces for global land areas. Int. J. Climatol..

[47] Escobar L.E., Lira-Noriega A., Medina-Vogel G., Peterson A.T. (2014). Potential for spread of the white-nose fungus (*Pseudogymnoascus destructans*) in the Americas: Use of Maxent and NicheA to assure strict model transference. Geospat. Health.

[48] Datta A., Schweiger O., Kühn I. (2020). Origin of climatic data can determine the transferability of species distribution models. NeoBiota.

[49] Title P.O., Bemmels J.B. (2018). ENVIREM: An expanded set of bioclimatic and topographic variables increases flexibility and improves performance of ecological niche modeling. Ecography.

[50] Tytar V.M., Baidashnikov O. (2021). Associations between habitat quality and body size in the Carpathian land snail *Vestia turgida*: Species distribution model selection and assessment of performance. Zoodiversity.

[51] Chadaeva V., Pshegusov R. (2022). Identification of degradation factors in mountain semiarid rangelands using spatial distribution modelling and ecological niche theory. Geocarto Int..

[52] Amatulli G., Domisch S., Tuanmu M.N., Parmentier B., Ranipeta A., Malczyk J., Jetz W. (2018). A suite of global, cross-scale topographic variables for environmental and biodiversity modeling. Sci. Data.

[53] Leemans R., De Groot R.S. (2005). Millennium Ecosystem Assessment. Ecosystems and Human Well-Being.

[54] Pearson R.G., Dawson T.P., Liu C. (2004). Modelling species distributions in Britain: A hierarchical integration of climate and land-cover data. Ecography.

[55] Chauvier Y., Thuiller W., Brun P., Lavergne S., Descombes P., Karger D.N., Renaud J., Zimmermann N.E. (2021). Influence of climate, soil, and land cover on plant species distribution in the European Alps. Ecol. Monogr..

[56] Tuanmu M.-N., Jetz W. (2014). Consensus land cover. Glob. Ecol. Biogeogr..

[57] Domisch S., Amatulli G., Jetz W. (2015). Near-global freshwater-specific environmental variables for biodiversity analyses in 1 km resolution. Sci. Data.

[58] Gage H., Dayton L., Fitzgerald A. (2006). Habitat suitability models for desert amphibians. Biol. Conserv..

[59] Roe N.A., Ducey M.J., Lee T.D., Fraser O.L., Colter R.A., Hallett R.A. (2022). Soil chemical variables improve models of understorey plant species distributions. J. Biogeogr..

[60] Shangguan W., Dai Y., Duan Q., Liu B., Yuan H. (2014). A global soil data set for earth system modeling. J. Adv. Model. Earth Syst..

[61] Braunisch V., Coppes J., Arlettaz R., Suchant R., Schmid H., Bollmann K. (2013). Selecting from correlated climate variables: A major source of uncertainty for predicting species distributions under climate change. Ecography.

[62] Leroy B., Meynard C.N., Bellard C., Courchamp F. (2016). ‘virtualspecies’: An R package to generate virtual species distributions. Ecography.

[63] Buse J., Griebeler E.M. (2011). Incorporating classified dispersal assumptions in predictive distribution models—A case study with grasshoppers and bush-crickets. Ecol. Model..

[64] Guisan A., Thuiller W., Zimmermann N. (2017). Habitat Suitability and Distribution Models: With Applications in R (Ecology, Biodiversity and Conservation).

[65] Puschendorf R., Carnaval A.C., VanDerWal J., Zumbado-Ulate H., Chaves G., Bolaños F., Alford R.A. (2009). Distribution models for the amphibian chytrid *Batrachochytrium dendrobatidis* in Costa Rica: Proposing climatic refuges as a conservation tool. Divers. Distrib..

[66] Carlson C.J. (2020). ‘embarcadero’: Species distribution modelling with Bayesian additive regression trees in R. Methods Ecol. Evol..

[67] Kursa M.B., Jankowski A., Rudnicki W.R. (2010). Boruta—A system for feature selection. Fundam. Inform..

[68] Velazco S.J.E., Rose M.B., Andrade A.F.A., Minoli I., Franklin J. (2022). flexsdm: An R package for supporting a comprehensive and flexible species distribution modelling workflow. Methods Ecol. Evol..

[69] Allouche O., Tsoar A., Kadmon R. (2006). Assessing the accuracy of species distribution models: Prevalence, kappa and the true skill statistic (TSS). J. Appl. Ecol..

[70] Lobo J.M., Jimenez-Valverde A., Real R. (2008). AUC: A misleading measure of the performance of predictive distribution models. Glob. Ecol. Biogeogr..

[71] Boyce M.S., Vernier P.R., Nielsen S.E., Schmiegelow F.K.A. (2002). Evaluating resource selection functions. Ecol. Model..

[72] Hirzel A.H., Le Lay G., Helfer V., Randin C., Guisan A. (2006). Evaluating the ability of habitat suitability models to predict species presences. Ecol. Model..

[73] Angelieri C.C., Adams-Hosking C., Ferraz K.M., de Souza M.P., McAlpine C.A. (2016). Using species distribution models to predict potential landscape restoration effects on puma conservation. PLoS ONE.

[74] Waltari E., Guralnick R.P. (2008). Ecological niche modeling of montane mammals in the Great Basin, North America: Examining past and present connectivity of species across basins and ranges. J. Biogeogr..

[75] Conrad O., Bechtel B., Bock M., Dietrich H., Fischer E., Gerlitz L., Wehberg J., Wichmann W., Böhne J. (2015). System for automated geoscientific analyses (SAGA) v. 2.1.4. Geosci. Model Dev. Discuss..

[76] Hammer Ø., Harper D.A.T., Ryan P.D. (2001). PAST: Paleontological statistics software package for education and data analysis. Palaeontol. Electron..

[77] R Core Team 2020 (2020). R: A Language and Environment for Statistical Computing.

[78] Driscoll D.M., Fong J.M.Y. (1992). Continentality: A basic climatic parameter reexamined. Int. J. Climatol..

[79] Ron S.R. (2005). Predicting the distribution of the amphibian pathogen *Batrachochytrium dendrobatidis* in the New World. Biotropica.

[80] Thorpe C.J., Lewis T.R., Fisher M.C., Wierzbicki C.J., Kulkarni S., Pryce D., Davies L., Watve A., Knight M.E. (2018). Climate structuring of *Batrachochytrium dendrobatidis* infection in the threatened amphibians of the northern Western Ghats, India. R. Soc. Open Sci..

[81] Olson D.H., Ronnenberg K.L., Glidden C.K., Christiansen K.R., Blaustein A.R. (2021). Global patterns of the fungal pathogen *Batrachochytrium dendrobatidis* support conservation urgency. Front. Vet. Sci..

[82] Piotrowski J.S., Annis S.L., Longcore J.E. (2004). Physiology of *Batrachochytrium dendrobatidis*, a chytrid pathogen of amphibians. Mycologia.

[83] López M., Rebollar E.A., Harris R.N., Vredenburg V.T., Hero J.-M. (2017). Temporal variation of the skin bacterial community and *Batrachochytrium dendrobatidis* infection in the terrestrial cryptic frog *Philoria loveridgei*. Front. Microbiol..

[84] Esser L.F. (2021). The 19 Bioclimatic Variables. https://luizfesser.wordpress.com/2021/03/08/the-19-bioclimatic-variables/.

[85] Cervellini M., Zannini P., Di Musciano M., Fattorini S., Jiménez-Alfaro B., Rocchini D., Field R., Vetaas O.R., Irl S.D., Beierkuhnlein C. (2020). A grid-based map for the Biogeographical Regions of Europe. Biodivers. Data J..

[86] Buckley L.B., Jetz W. (2007). Environmental and historical constraints on global patterns of amphibian richness. Proc. Biol. Sci..

[87] Sun G., Domec J.-C., Amatya D.M., Amatya D.M., Williams T.M., Bren L., de Long C. (2016). Forest evapotranspiration: Measurement and modelling at multiple scales. Forest Hydrology: Processes, Management and Assessment.

[88] James T.Y., Toledo L.F., Rödder D., Leite D.S., Belasen A.M., Betancourt-Román C.M., Jenkinson T.S., Soto-Azat C., Lambertini C., Longo A.V. (2015). Disentangling host, pathogen, and environmental determinants of a recently emerged wildlife disease: Lessons from the first 15 years of amphibian chytridiomycosis research. Ecol. Evol..

[89] Davidson C., Williamson C., Vincent K., Simonich S., Yip K., Hero J.M., Kriger K. (2013). Anuran population declines occur on an elevational gradient in the Western Hemisphere. Herpetol. Conserv. Biol..

[90] Li Z., Qi W., Keping S., Jiang F. (2021). Prevalence of *Batrachochytrium dendrobatidis* in amphibians from 2000 to 2021: A global systematic review and meta-analysis. Front. Vet. Sci..

[91] Robinson C.W., Mcnulty S.A., Titus V.R. (2018). No safe space: Prevalence and distribution of *Batrachochytrium dendrobatidisin* amphibians in a highly-protected landscape. Herpetol. Conserv. Biol..

[92] Kriger K.M., Hero J.-M. (2007). The chytrid fungus *Batrachochytrium dendrobatidis* is non-randomly distributed across amphibian breeding habitats. Divers. Distrib..

[93] Blooi M., Laking A.E., Martel A., Haesebrouck F., Jocque M., Brown T., Green S., Vences M., Bletz M.C., Pasmans F. (2017). Host niche may determine disease-driven extinction risk. PLoS ONE.

[94] Jourgholami M., Karami S., Tavankar F., Lo Monaco A., Picchio R. (2021). Effects of slope gradient on runoff and sediment yield on machine-induced compacted soil in temperate forests. Forests.

[95] Reeves R.A., Pierce C.L., Vandever M.W., Muths E., Smalling K.L. (2017). Amphibians, pesticides, and the amphibian chytrid fungus in restored wetlands in agricultural landscapes. Herpetol. Conserv. Biol..

[96] Davidson C., Benard M.F., Shaffer H.B., Parker J.M., O’Leary C., Conlon J.M., Rollins-Smith L.A. (2007). Effects of chytrid and carbaryl exposure on survival, growth and skin peptide defenses in foothill yellow-legged frogs. Environ. Sci. Technol..

[97] Brannelly L.A., Richards-Zawacki C.L., Pessier A.P. (2012). Clinical trials with itraconazole as a treatment for chytrid fungal infections in amphibians. Dis. Aquat. Org..

[98] Hansen N.A., Scheele B.C., Driscoll D.A., Lindenmayer D.B. (2019). Amphibians in agricultural landscapes: The habitat value of crop areas, linear plantings and remnant woodland patches. Anim. Conserv..

[99] Tyler M.J., Wassersug R., Smith B. (2007). How frogs and humans interact: Influences beyond habitat destruction, epidemics and global warming. Appl. Herpetol..

[100] Tytar V., Nekrasova O., Pupins M. (2019). Positive Relationships Between Human Impact and Biodiversity: The Case of the Fire-Bellied Toad (*Bombina bombina*) in Europe. Proceedings of the 12th International Scientific and Practical Conference “Environment. Technology. Resources”.

[101] Kurylenko V.G., Verves Y.A. (1999). Amphibians and Reptiles of Ukraine.

[102] Cohen J.M., Civitello D.J., Brace A.J., Feichtinger E.M., Rohr J.R. (2016). Spatial scale modulates the strength of ecological processes driving disease distributions. Proc. Natl. Acad. Sci. USA.

[103] Alvarado-Rybak M., Lepe-Lopez M., Peñafiel-Ricaurte A., Valenzuela-Sánchez A., Valdivia C., Mardones F.O., Bacigalupe L.D., Puschendorf R., Cunningham A.A., Azat C. (2021). Bioclimatic and anthropogenic variables shape the occurrence of *Batrachochytrium dendrobatidis* over a large latitudinal gradient. Sci. Rep..

[104] Fuchs A.J., Gilbert C.C., Kamilar J.M. (2018). Ecological niche modeling of the genus *Papio*. Am. J. Phys. Anthropol..

[105] Berger L., Roberts A.A., Voyles J., Longcore J.E., Murray K.A., Skerratt L.F. (2016). History and recent progress on chytridiomycosis in amphibians. Fungal Ecol..

[106] Lips K.R. (2016). Overview of chytrid emergence and impacts on amphibians. Philos. Trans. R. Soc. B.

